# Editorial Comment to Systemic treatment for coexisting mucinous urethral adenocarcinoma and prostate adenocarcinoma

**DOI:** 10.1002/iju5.12218

**Published:** 2020-09-04

**Authors:** Shogo Watari, Takuya Sadahira

**Affiliations:** ^1^ Department of Urology Dentistry and Pharmaceutical Sciences Okayama University Graduate School of Medicine Okayama Japan

The coexistence of mucinous urethral and prostate adenocarcinoma is challenging to diagnose because of its rarity. Nezu *et al*. reported a case of coexisting mucinous urethral and prostate adenocarcinoma managed with systemic treatment.^1^ They initially diagnosed a patient with prostate adenocarcinoma. However, hormone therapy and standard chemotherapy for prostate adenocarcinoma were ineffective. Re‐biopsy showed mucinous adenocarcinoma, and they changed the hormone drug to a colon cancer regimen, followed by a bladder cancer regimen. Despite their best efforts, the patient died 14 months after clinical presentation. Autopsy implied that epithelial‐mesenchymal transition had occurred in the metastatic lesions of the urethral adenocarcinoma.

Mucinous urethral adenocarcinoma has no standard treatment approach. The authors suggested that erlotinib might be effective because epidermal growth factor receptor (EGFR) immunostaining was strongly positive in this case. Bryce *et al*. reported a case of mucinous urethral adenocarcinoma in which targetable EGFR amplification led to successful treatment with erlotinib.^2^ Before erlotinib, the patient received both traditional bladder cancer and colon cancer regimens. However, the bladder cancer regimen led to only a brief response with severe side effects, and the colon cancer regimen had similar toxicity. Targeted therapy of erlotinib was effective for a full year and provided the highest quality of life for the patient. Genomic tumor analysis would offer a tool to provide precise data to guide the appropriate treatment of these rare cases. EGFR can be tested for in future mucinous urethral adenocarcinoma cases to inform treatment decisions.

## Conflict of interest

The authors declare no conflict of interest.
